# Youth solving pandemics: hopeful futures in Maths Claesson’s novel *Pandemic*

**DOI:** 10.1007/s11059-021-00608-8

**Published:** 2021-11-08

**Authors:** Helene Ehriander, Michael Godhe

**Affiliations:** 1 Linnæus University, Växjö, Sweden; 2 Linköping University, Linköping, Sweden

**Keywords:** Pandemic, Y/A speculative fiction, Hope, Environmental crises

## Abstract

Are representations of pandemics in fiction always bleak dystopian tales understood as nature’s revenge on the modern Faustian man, or could they also express hope and expand our imagination in a time of environmental crisis? In this article, we analyse the young adult novel *Pandemic* (Swedish title: *Pandemi*, 2018) by Swedish author Maths Claesson. *Pandemic* is the third novel in a trilogy (2013–2018) with 15-year-old astronaut-trainee Linux as the main protagonist. During his astronaut program on a space station, a pandemic breaks out on Earth. While scientists on Earth struggle to isolate the virus and find a vaccine, Linux and his fellow astronaut-trainees are asked by the WHO to try out a simulation, a computer game aimed at isolating a pandemic outbreak and finding a vaccine. Their simulation is successful and eventually becomes decisive for the solution of the current pandemic crisis on Earth. Departing from Critical Future Studies (Goode and Godhe, Cult Unbound J Curr Cul Res 9(1):108–129, 2017), we focus on the *figures of hope* (cf. Moylan, Demand the impossible: Science fiction and the utopian imagination, Methuen, pp. 1–2, 1986) for a sustainable future and analyse how the novel is widening the scopes of possible futures. We show how the computer simulation and the successful solution of the crisis serves as a vehicle for a broader discussion about what kind of future we want, a future where the conquest of space offers new opportunities, e.g. for solving the environmental crisis. While normally in Y/A speculative fiction, technology is almost exclusively depicted as ostensibly serving human needs, in *Pandemic* it is thanks to technology, and the younger generation’s particular skills, that the disease is conquered. In this sense, the novel is hopeful since it depicts the younger generation as being capable of developing different thinking patterns from those of the adult society.

Are representations of pandemics in fiction always bleak dystopian tales understood as nature’s revenge on the modern Faustian man? Or could they also express hope and expand our imagination in a time of environmental crisis? In this article, we analyse the young adult novel *Pandemic* (Swedish title: *Pandemi*, 2018) by Swedish author Maths Claesson. Departing from Critical Future Studies (Godhe & Goode, 2018; Goode & Godhe, 2017), we focus on the *figures of hope* (cf. Moylan, 1986, pp. 1–2) and analyse how the novel is widening the scopes for possible sustainable futures. As we will show in this article, the novel differs in many ways from traditional speculative fiction on viruses and ends with a fragile hope for humanity.

*Pandemic* is the third novel in a trilogy. In the first novel, *Uttagningen* (The selection, 2013), the main protagonist, Linux Svensson, spends his time daydreaming. Linux is a 15-year-old boy with a yearning to become an astronaut, and he signs up for a space ambassador contest for youths. After learning to stop daydreaming and to work hard with his studies and going through a cut-throat selection process, he is one of the few chosen for an astronaut-training program. In the second novel, *Kristallstaden* (The Crystal City, 2014), Linux is undergoing the space trainee program together with his new friends on a space station orbiting Earth. At the end of the novel, a pandemic breaks out on Earth. Linux and his friends are reassured that mass vaccination is going to take place, but, until then, Earth is set in quarantine.

In the third novel, *Pandemic*, scientists on Earth try to isolate the virus and find a vaccine. At the same time, together with youths from Nairobi, Toronto, Chennai, and other mega-cities, as well as the space cities orbiting Earth, Linux and his fellow astronaut-trainees are asked by the WHO in cooperation with game developer Genesis Z to do a beta-test of the simulation-game Viruz H3N2 in collaboration with an Artificial Intelligence (AI) being in order to identify software bugs and other problems. The simulation is eventually successful, and the youth players are told that the game has followed the real development of the pandemic outbreak on Earth. The result of the simulation is decisive for the solution of the current pandemic crisis on Earth.^1^ This is similar to the plot in Orson Scott Card’s seminal classic *Ender’s Game* (1991), but, in other ways, *Pandemic* is very different from Card’s novel.

In this article, we claim that the computer simulation and its successful result serves as a vehicle for a broader discussion of what kind of future we want to have. Departing from the interdisciplinary field Critical Future Studies (CFS), we focus on the *fragile hope* for humanity represented by the youth in Claesson’s narrative and how the novel is widening the scopes for possible sustainable futures rather than limiting our imagination. CFS is both a deconstructive and reconstructive theoretical and methodological enterprise, and popular cultures are a “rich repository of imaginative futurescapes” in the CFS perspective (Goode & Godhe, 2017, p. 111). CFS investigates “the ways in which cultural texts not only represent the future, but also actively shape it by opening up or closing down imaginative possibilities” (Godhe & Goode, 2018, p. 151). In this sense, CFS aims to contribute to a futural public sphere and “challenge a prevalent contemporary cynicism about our capacity to imagine alternative futures while trapped in a parlous present.” In this case, we are interested in how the story of Linux opens up particular congealed imaginaries and problematize them (Goode and Godhe, p. 109).

*Pandemic* is a science fiction novel comprising also other generic elements from teen noir, critical utopias and critical dystopias, contemporary popular fiction, games, and movies. To define science fiction is of course an almost impossible enterprise (Määttä, 2006, 2008), but, with inspiration from Carl Freedman (2000), we define science fiction as a representational form where the generic tendency is cognitive estrangement. We use the term “representational *form*,” since it includes both textual, aural, and visual genres, forms and hybrids, and it is important to remember that almost no science fiction product is pure cognitive estrangement. There are always other forms, although not dominant, present in the narrative, such as fantasy, horror, and so on.

In the following, we analyse how Linux and some of his astronaut-trainee friends in *Pandemic* (Tuva from Sweden, Ahmed from Denmark, Pierre from France, Johánn from Iceland, and Sinéad from Ireland, to mention a few of them) find the solution for the pandemic crisis by contesting aetonormativity. Aetonormativity is a power relation common in children’s literature, meaning “adult normativity that governs the way children’s literature has been patterned from its emergence until the present day” (Nikolajeva, 2009, p. 16). We show how using the future in the narrative for mirroring the present makes it possible to contest aetonormativity and expand the repertoire of possible futures. Subsequently, we discuss how the narrative in the trilogy is using historical time in setting the futurescape—namely, a future loaded with references to our current times and stating that our problems consist not only of threats in the form of pandemics. Our contemporary crises are pointed out through the narrative and urge us to expand our imagination and think in new and innovative ways. Subsequently, we discuss how some of the tropes recurring in outbreak narratives (Schweitzer, 2018, pp. 38-69) are used or reinterpreted in *Pandemic*.

In *History and the Contemporary Novel*, David Cowart discusses the historical novel. Cowart claims that every narrative with a conscious approach to history concerning plot, characters, or environment are in some sense historical. One of the categories in Cowart’s scheme is “The Way It Will Be—fictions whose authors reverse history to contemplate the future” (Cowart, 1989, pp. 8–10). It comprises stories that are using historical time for mirroring our contemporary society, and this is the case in the Linux trilogy, where our present time is the past for Linux and his friends. This is also how speculative genres such as science fiction, critical utopias, and critical dystopias work by setting the plot in the future for discussing contemporary issues—how we live today and the possible consequences of our actions, our science, and our technology (cf. Godhe & Ramsten, 2010; Suvin, 1979). This is especially valid in disaster narratives (e.g., pandemics or environmental degradation), which are closely connected with problems caused by technological and scientific development, and current social trajectories. But the Linux trilogy differs in the sense that science and technology, exemplified by the computer simulation and the AI being working together with the youths, are tools for solving our contemporary crises—as we will discuss below. There is no case of a Frankenstein complex (McCauley, 2007) here in forms of, for example, an AI uprising; nor are there any catastrophes caused by technological or scientific hubris. There seems to be an upcoming trend in Y/A science fiction that young people sociably and wisely work together with humanlike AI-robots to solve problems in the future (see Lee Bacon’s novel *The Last Human*, 2019).

In *Pandemic*, contesting aetonormativity is the necessary means to save the world from the plague, but also to expand the scopes for different and sustainable futures where the fragile hope for humanity’s salvation is connected to the youth. The contesting of aetonormativity, moreover, is made explicit by the youths playing a simulation of a pandemic outbreak, collaborating with an AI companion. In the beginning of the astronaut program, the young disciples often relax in their spare time by playing computer games—something a young reader is very well acquainted with. The youth players save the world by playing a computer simulation of a pandemic outbreak. They have learned to use technology in constructive and innovative ways through their computer gaming, to develop strategic thinking, and to find new solutions—maybe because they believe it is just game and that they have nothing to lose except a ‘game over’?

In Y/A speculative fiction, technology is almost exclusively depicted as ostensibly serving human needs. Children’s and juvenile science fiction stories rarely take place in a future where technology and science are supporting elements. Most companions and readers mention Jules Verne’s novels and other books originally written for an adult audience but also read by a youth audience: for example, Michael Crichton’s *Jurassic Park* (1990) (cf. Yates, 1996). One can also mention Robert A. Heinlein’s juveniles in the 1940s and 1950s (Mulcahy, 1997), where technology and science have a decisive role in the narrative, but this is an exception.

The success with the computer simulation is obviously a triumph for the kids (and the readers) since the adult research community has questioned the children’s ability to find ways to isolate the virus and discover a vaccine. One researcher thinks that computer games are not good for children (Claesson, 2018, p. 734): “You forget your homework and forget to sleep and spend the whole night awake, even though it is school the next day. And some of you are having trouble distinguishing between what is real or not” (”Ni glömmer läxor och glömmer att sova och sitter uppe hela nätterna, trots att det är skola dagen därpå. Och några av er får svårt att skilja på vad som är verkligt och inte”).^2^ As Maria Nikolajeva (2009, p. 18) remarks,

In dystopian novels for children, the adult world is interrogated, as it is presumably the adults who have created the highly ordered, hierarchical, but dull society that serves as a setting for a dystopian plot. In fact, such an interrogation is one of the many stereotypes of the dystopian novel for young readers. Further, in accordance with the conventions of children’s literature, it is the child or a young person who questions the society and reaches the insight about its injustice, but it is the adult society that suppresses the revolt.

Although it is not quite a revolt in the literal sense suppressed by the adult society, the experts in *Pandemic* first refuse to accept the youths’ contribution and their solution to the problem. They are diminishing, condescending, didacticizing, and dismissive. One of the players, Ahmed Mohamad from Copenhagen, comes up with ideas for isolating the virus, but the scientists call him a “little fella” twice and dismiss his ideas as rubbish. When he has some trouble explaining his different and ingenious ideas, he is told that they, the youth, “are disturbing important research with your nonsense” (Claesson, 2018, p. 744 [“stör viktig forskning med era dumheter”]). And furthermore,

You must understand one thing, my stubborn little fella. The best and foremost experts in the solar system on immunology and virus diseases are here in our laboratory. […] You cannot seriously believe that you know something they would not have thought of a long, long time ago? (Claesson, 2018, p. 745) [”Du måste förstå en sak, min envise lille vän. Här i laboratoriet finns solsystemets främsta experter på immunologi och virussjukdomar. […] Du kan inte på allvar tro att du vet något som de inte tänkt på för länge, länge sedan?”])

When the adult society struggles in vain to find strategies for isolating the virus and construct an efficient vaccine, Ahmed—together with Linux as a sidekick and supported by the AI companion and the other players—saves humanity from the pending catastrophe. He can think outside the box, is humble, and can cooperate and think globally. He uses his experiences from computer gaming as well as from scientific literature to find solutions. When the pompous scientific community tries to develop a vaccine, Ahmed suggests that they replace the vaccine with antibodies from a Rwandan woman who has recovered from the virus. Furthermore, he suggests that they use bio-nanorobots to steer the antibodies the right way if the virus attacks. Eventually, he manages to convince the scientists.

The youths in the novel represent the problem solvers and the fragile hope for humanity. Following Nikolajeva (2009, p. 18), an “aetonormative reading of dystopian novels leaves one with quite pessimistic conclusions. The depiction of childhood in conventional, or utopian, children’s fiction reflects the adult writers’ view, often highly idealized, sometimes nostalgic.” Since the narrative in the Linux trilogy is a hybrid with both dystopian and utopian elements, it is perhaps more valid with the young reader’s own experience. According to Nikolajeva (2009, p. 18), which in her opinion is a paradox, “the vision of the future in children’s novels is […] likely to be radically different from the ideas young people today may have of their own future.” In *Pandemic*, the children are expanding our imagination, finding the solution, and they are cooperating across all borders. By contesting adult values “leaking through the young protagonists’ deliberately naïve perspective” (Nikolajeva, 2009, p. 18), Maths Claesson’s narrative is more congenial with young readers visions of the future.

In our view, Claesson’s text is also co-producing the young readers voice with “the reflection of our own fears and our own feeling of guilt” (Nikolajeva, 2009, p. 18). One interpretation here is that the threat in form of a pandemic is the result of the adult’s unsustainable ways of living. Even if the adults once have ruined many parts of Earth (although the narrative does not clearly state when this happened), they appear to be more responsible and conscious about the environment. It is the youth, however, who represent a different way of proceeding into the future. The WHO and the scientist change their opinions and describe how new ways of thinking (explored by the youths) result in unexpected solutions to gigantic problems. The adult society apologizes, and the Secretary-General of UN salutes Ahmed (Claesson, 2018, p. 780): “Never has so many had one single person to thank for so much” (“Aldrig har så många haft en enda person att tacka för så mycket”). By paraphrasing Winston Churchill’s famous speech in the House of Commons, 4 June 1940, the Secretary-General signals that Ahmed’s solution to the problem is a historic event of great importance for humanity.

In this sense, *Pandemic* is different from common Y/A tales of emerging catastrophes. Science fiction is not a particularly common genre in children’s and young people’s literature. In many ways, it is a problematic genre that clashes with the traditional perceptions of what a children's book should be like. One of the basic rules in children’s literature is that a story must not discourage the young reader’s hope and that there must be something that can be called a *happy ending*. There is thus a difference between a thrilling space adventure where the protagonists return to Earth as heroes, and a globe plagued by environmental destruction forcing humanity to colonize outer space. In recent years, several dark and frightening dystopias for younger readers have been published, and although there is hope in the narratives, it is frightening to imagine a third world war, nuclear weapons, plagues, and climate change. Children nowadays are aware of how the world is shaped and the threats we are facing, and the obvious example of this is the famous Swedish climate activist Greta Thunberg. In *Pandemic*, Claesson balances between dystopia and utopia; the Earth has collapsed and there is a scarcity of resources, but humanity has finally taken responsibility for the situation it created and has started to change direction. The story depicts visions of a new and sustainable way of life, which the children believe is better than how it was before. It is a critical utopia (cf. Moylan, 1986) with a fragile hope for solving many of the problems we are facing, as it depicts the younger generation as the inheritors of progress and, consequently, as the problem solvers of tomorrow (cf. Godhe, 2003). There is no return to the status quo when the novel finishes, neither for the protagonist nor the readers. Rather, we must learn to live in a world of impending risks, pointed out in the use of historical time in the narrative, urging us to change our contemporary trajectories. This also became obvious in the use of the futurescape in the narrative.

The futurescape of Linux’ hometown, Stockholm, is depicted as crowded. Overpopulation has forced humanity to build cities in space when it is not possible to build more skyscrapers. Claesson avoids explaining too much of the plot and the background for the readers (a ‘don’t tell me, show me!’ method), which is otherwise common in many speculative fiction plots, by the author or a voice over (in television or film) retelling the events that eventually caused the catastrophe. The backdrop for the novels is instead narrated through references to global climate change, altered food habits (people eat less meat except for bugs and insects), and so on, but this is not something with which the characters are obsessed. They do not explain how and why the world has changed for good or worse but leave that to the reader’s imagination.

With a homodiegetic narrator, representations of the environment are affected. Since *Pandemic* is set in the future, there are subtle reflections from the main protagonist Linux, concerning changes in society, for example: “It is only desert in Australasia anyway” (Claesson, 2018, p. 9 [“Det är ju bara öken i Australasien ändå”]). Our knowledge of environmental change is supplemented through the eyes of Linux. And through the dialogue between Linux and his friends, we learn that Stockholm in the future is much warmer and that several places on Earth are uninhabitable due to global warming. When the kids play the simulation of the virus’ global spread, it is almost uncanny how much it resembles our current Covid-19 crisis.

Pandemics in literary and visual speculative fiction texts are common, be it post-apocalyptic fantasy, science fiction, horror, or other genres, from Mary Shelley’s *The Last Man* (1826) to *The Walking Dead* (broadcasted from 2010 onward) (cf. Määttä, 2015). According to Dahlia Schweitzer (2018, pp. 38-69), there are six basic tropes in all outbreak narratives. These tropes can apply to the Linux trilogy and they also help to further expose some of the explicit and implicit criticism in *Pandemic*, pointing to the fact that the crisis concerns much more than a globally spread virus. It might be the case, according to the narrative, that the degradation of the environment in our industrial society and the rise of instrumental reason and human exceptionalism (cf. Anderson & Perrin, 2018) have in fact caused the virus. The virus is thus a symptom of unsustainable ways of living.

*The accident* is the trope starting the chain of events (Schweitzer, 2018, p. 42). The spread of Virus H3N2 in *Pandemic* starts with a flood catastrophe in Bangladesh and the first casualties of the virus. The computer simulation is constructed and developed by the game developer Genesis Z and based on data from the earlier spread of pandemics, among them the Spanish flu, avian flu, and SARS. The WHO has shared information from their data base, and the game is extremely realistic.

The second trope, “Othering,” concerns a split between us and them, and the accident is transformed to a threat to us (Schweitzer, 2018, p. 44). A scapegoat is constructed (them), and in *Pandemic* the virus spreads from Asia (East) to Europe (West). This is a cultural threat, but it is also a global problem since the flood might be caused by climate change. The alien is the dangerous and scary—the people in Bangladesh are either victims or the ones to blame for the spread of the disease (cf. Schweitzer, 2018, p. 81). Another example is the German character Hugo Beckenbauer in the simulation. Hugo travels from Frankfurt to Chennai, India, where he is visiting tourist destinations, restaurants, hotels, and a yoga resort. He also visits a bird market with thousands of rare and tame wild birds, and by his acting in this matter, he illuminates how an infectious disease is spread from an animal to a human (zoonosis). Hugo epitomizes the Western middle-class tourist. As one of the simulation players emphasizes,

There are always people prepared to pay huge amounts of money in order to brag that they have a rare bird on the roof of the house where they are living. Due to the flood it was extraordinary many birds on the market, pigeonholed randomly in far too narrow cages” (Claesson, 2018, p. 684 [”Det finns ju människor som är beredda att betala stora summor pengar för att skryta med att de har en sällsynt fågel på taket på huset där de bor. På grund av översvämningen var det ovanligt många fåglar på marknaden, instoppade huller om buller i alldeles för trånga burar”]).

There is also a photograph of Hugo with a parrot on his shoulder. The morning before he leaves Chennai, he eats breakfast at the hotel and incidentally coughs and infects a 17-year-old football player from China. And she brings the virus with her on her way home and eventually dies due to breathing problems. Hugo, in turn, dies on the flight back to Frankfurt, as do one of the stewardesses and some passengers sitting close to him. The episode is interesting because it blurs the ‘us’ and ‘them’ trope. We do not know for sure, as one of the simulation players states, if Hugo was infected at the bird market, but it is likely that it happened there. In this case, the spread of the virus is global and concerns the whole of humanity. The episode also highlights horrible animal industrial agricultures in different parts of the world and how consumption helps to keep them going. Hugo represents Western lifestyles with tourism, environmental degradation, and the exploitation of nature and animals. So, it is both a local and structural problem, but human exceptionalism seems to be the main cause, along with poverty in parts of the world where the big animal markets are located. The simulation episode is also interesting since it contests a racist trope, that “the ‘colored’ body of the colonized was constructed as the dark source of infection, pollution, disorder, etc. that threatened to overwhelm white manhood (cities, civilization, the family, the white personal body)” (Schweitzer, 2018, p. 44). As we mentioned above, it is a black woman helping the youth to save the world from the plague by offering samples of her antibodies. In the simulation, it is a white man spreading the disease.

From a CFS perspective, this is a question of expanding our imagination by contesting aetonormativity. This is obvious when the headmaster is addressing the space-trainee pupils on the space station at the beginning of the crisis and tells them that a pandemic haunts Earth in large parts of the world—even Europe—but that he also has good news:

Researchers and doctors have been working around the clock and they have managed to produce a vaccine. So, we have an efficient weapon, and we can stop the spread of the virus. In all continents and countries, mass vaccination never seen before are prepared for. The problem is that it will take time to distribute it to all people all over the world and make sure that everyone is getting vaccinated. But eventually we will defeat the disease. (Claesson, 2018, pp. 572–573 [Forskare och läkare har jobbat dygnet runt och lyckats framställa ett vaccin. Vi har därmed ett effektivt vapen och kan förhindra vidare spridning av infektionssjukdomen. I alla världsdelar och länder förbereds därför en massvaccination av aldrig förut skådat slag. Problemet är att det tar tid att tillverka sådana enorma mängder vaccin, det tar tid att fördela det över världen och se till att alla blir vaccinerade. Men till slut kommer vi att besegra sjukdomen]).

This is a typical aetonormative way of addressing the pupils. The adult society has the solution, and the solution is a vaccine. And although the headmaster’s speech includes all continents and countries, still social injustice, poverty, and the lack of a global Welfare State or community make part of the world more vulnerable to the pandemic.

A vaccine is of course necessary, but it is the symptom the adult world emphasizes, not the cause. If animal husbandry, breeding, and transport are not changed, then there is an impending danger that new pandemics will afflict humanity. As the character Jamie says to Ahmed, “Bubonic plague and swine flu instead, or those rabid bats you mentioned, they sound really interesting” (Claesson, 2018, p. 725 [“Böldpest och svinpest i stället, eller de där rabiesfladdermössen du pratade om, de lät ju intressanta”]). In *Pandemic*, there are some signs that this will change, however, once again represented by the youth. In the future setting of the novel, people still eat meat but mostly cultivate insects. In one episode, the pupils discuss the old and abandoned habit of eating pigs on Christmas and how other animals such as cows, bulls, and calves were also slaughtered and parcelled for consumption. The character Sinéad finds it absurd to eat anything with a consciousness:

“I’m going to *throw up*,” Sinéad said, with a pale face. “I don’t think you should eat anything that thinks.”

“Our world is different,” Linux said.

“Yes,” Sinéad said. “Different. And better” (Claesson, 2018, p. 603 [“Jag *kräks*,” sade Sinéad, vit i ansiktet. “Jag tycker inte att man ska äta något som tänker.” / “Vår värld är annorlunda,” sade Linux. / “Ja,” sade Sinéad. ”Annorlunda. Och bättre”]).

The third trope in outbreak narratives concerns how different kinds of boundaries create a sense of security (Schweitzer, 2018, p. 47). In *Pandemic*, the headmaster and the WHO claim that they have the pandemic under control, but the virus soon becomes hard to isolate. This is also a feature of the Enlightenment and the idea of progress: the belief in our ability to control nature (Levin, 2000). Once again, it is the youth solving the problem of how to isolate the virus. After getting in touch with the immunologist doctor Rieux (an intertextual reference to the doctor in Albert Camus’ 1947 *La Peste*), Linux and his fellow players take measures to limit the virus’s impact. These measures are all too well known by us now—lockdown: stop travelling, work from home, close down kindergartens and schools, allow no spectators at football games and concerts, close down malls and restaurants, and let people order groceries and other life necessities from the Net. Once again, technology is serving the youth’s solutions: “People have to order from home and let bots and drones take care of the deliveries. Is there anything missing, they can use the 3D printer and manufacture what they need” (Claesson, 2018, p. 716 [“Folk får beställa hemifrån och låta botar och drönare sköta leveranserna. Saknas det något, kan de använda 3D-skrivaren och tillverka vad de behöver”]).

The fourth trope concerns collaboration and unity, and even though the pandemic “separates us, contagious disease can also bring us together” (Schweitzer, 2018, p. 49). No matter who you are or where you are, you can be infected, and it is obvious that collaboration and unity are needed to solve the problem, regardless of race, gender, class, and age. Concerning the fifth and sixth tropes in outbreak narratives, Claesson’s trilogy certainly deviates from them. The fifth trope is about making the invisible visible (Schweitzer, 2018, p. 50): anyone could carry the disease which makes it scary and threatening, but in *Pandemic* the virus is far away from the central characters, although in some sense visible through the computer simulation. The sixth trope is “fear of progress combined with the development of an integrated ‘risk society’” (Schweitzer, 2018, p. 54). It concerns the ideologically conservative context, where science, technology, progress, and globalization are contested. This is certainly not the case in *Pandemic*. The novel (in fact, the whole trilogy) sometimes resembles technological solutionism (cf. Taffel, 2018) but without falling into technophilicism, valuing global cooperation and solidarity instead.

As children’s literature scholar Maria Nilson (2013, pp. 134–138) states, the protagonist in dark Y/A literature is often the chosen one fighting against darkness and evil. This is also common in Y/A narratives on pandemics—for instance, Rory Power's debut (2019) *Wilder Girls*, where girls set in quarantine in a boarding school are uniting in female friendship in a broken-down society, braving the horrors in the fight against ‘Tox.’ This is a hybrid novel with features from dystopias, horror, and outbreak narratives, where the fragile hope lies in courage and female friendship.

In a sense, Linux and his friends are chosen, in this case for the astronaut programme due to their special personal qualities. They are smart, can cooperate and work together, and have good physiques and other attractive personal qualities, but, at the same time, they are complex characters with flaws and deficits. Ahmed, who is the driving force in finding the cure for the virus, is whimsical and absent-minded but has the capacity to find innovative solutions. Overall, the characters are plotted and carved out in way that Y/A readers can recognize, and the narrative, obviously in favour of the youth protagonists, contests aetonormativity.

From being a beta test for a virus simulation, the computer simulation becomes the symbol for collaboration and unity. The narrative also sheds light on other measures necessary in order to avoid future catastrophes—measures including a change in consumption, Western lifestyles, animal husbandry, and the breeding and transport of animals. In that sense, *Pandemic* is not exclusively a narrative about a virus threatening the whole world; it is a novel concerning our choice of the future we want. By contesting our social and cultural imaginaries, what we have taken-for-granted (cf. Godhe & Goode, 2018; Goode & Godhe, 2017), the novel expands our imagination and contests human exceptionalism through the fragile hope symbolized by the young protagonists. In a sense, *Pandemic* may be characterized as a novel representing what Terry Eagleton has labelled hope without optimism (Eagleton, 2015). Eagleton discusses different figures of hope, and optimism might represent stagnation because it signals the idea that everything eventually is going to be solved and turn out fine. A kind of pessimism (but not cynicism), on the other hand, urges us instead to act to solve the problem(s), even if we cannot know whether or not such action is enough or is in vain. The youth in *Pandemic* are certainly not naïve optimists, since there is no turning back to the old ways of living.

Judging from *Pandemic* and other science fiction novels mentioned in this article, current representations of plagues in fiction are not always bleak dystopian tales but rather express a fragile hope and expand our imagination in a time of environmental crisis. In children’s and Y/A literature, there are relatively few science fiction stories set in the future with technology and science as supporting elements. A problematical aspect of science fiction is that technological development is rapid, and it is difficult for writers to stay up to date with technical inventions and scientific achievements. What appears to be a distant future can be part of our everyday life in a few years. In that case, narratives speculating on what the future will look like can turn out to be incorrect and a bit comical. This is not the case, however, with Maths Claesson’s novel *Pandemic*. When he published it in 2018, a global pandemic was still not believed to be an instant threat to humanity, but the problems the story illustrates became reality much faster than anyone could have imagined. If earlier representations in many ways have been conservative or even technophobic (and this goes for many late modern science fiction narratives), technology and science are now in the service of humanity. In that sense, *Pandemic* recaptures a belief in progress, although conditioned, since it must be sustainable and cannot be at the expense of nature and other living beings.

## Footnotes

1 The first two novels in the Linux series were published as separate volumes. The third volume, *Pandemi* (*Pandemic*, 2018), also contains the first two parts of the trilogy. In this article, every reference is to the third volume (including our references to the first two novels).

2 All quotes from the Swedish novels are translated into English by Helene Ehriander and Michael Godhe.
